# The fundamental rights risks of countering cognitive warfare with artificial intelligence

**DOI:** 10.1007/s10676-025-09868-9

**Published:** 2025-10-06

**Authors:** Henning Lahmann, Bart Custers, Benjamyn I. Scott

**Affiliations:** Leiden University, Leiden, Netherlands

**Keywords:** Cognitive warfare, Disinformation, Artificial intelligence, Fundamental rights, Epistemic paternalism, Precautionary principle

## Abstract

This article analyses ideas to use AI-supported systems to counter ‘cognitive warfare’ and critically examines the implications of such systems for fundamental rights and values. After explicating the notion of ‘cognitive warfare’ as used in contemporary public security discourse, the article describes the emergence of generative AI tools that are expected to exacerbate the problem of adversarial activities against the online information ecosystems of democratic societies. In response, researchers and policymakers have proposed to utilize AI to devise countermeasures, ranging from AI-based early warning systems to state-run content moderation tools. These interventions, however, interfere, to different degrees, with fundamental rights and values such as privacy, communication rights, and self-determination. This article argues that such proposals insufficiently account for the complexity of contemporary online information ecosystems, particularly the inherent difficulty in establishing causality and attribution. Reliance on the precautionary principle might offer a justificatory frame for AI-enabled measures to counter ‘cognitive warfare’ in the absence of conclusive empirical evidence of harm. However, any such state intervention must be based in law and adhere to strict proportionality.

## Introduction

Reports about the harmful effects of disinformation online are ubiquitous, most frequently in connection with elections and other democratic decision-making processes (Colomina et al., 2021). Public fears about foreign interference by way of online influence campaigns grew after two unexpected and highly consequential political events in 2016: the referendum that determined the United Kingdom’s departure from the European Union (‘Brexit’) and the presidential election that brought Donald Trump to power in the United States. Most recently, both the parliamentary and the presidential elections in Slovakia were widely described as having been tainted by a contamination of the information ecosystem (Meaker, 2023; Hockenos, 2024). In December 2024, the Romanian Constitutional Court even annulled the first round of the country’s presidential election after intelligence surfaced a months-long influence campaign to boost the chances of an independent, nationalist and pro-Russian candidate (Selejan-Gutan, 2024). The common thread connecting these examples is an increasing involvement of foreign adversarial actors in the distribution of manipulative information in Western liberal democracies, in what has been framed as a rising prevalence of ‘cognitive warfare’ (NATO Allied Command Transformation).

Experts have started issuing warnings that the rise of AI technologies will exacerbate the challenge (Goldstein & Sastry, 2023). As for the examples above, AI-generated deepfakes – a piece of audio or audiovisual content that was digitally altered – were observed in the recent elections in both Slovenia and Romania (Vainaite, 2025). As these tools become more sophisticated and accessible, it is feared that they will further contribute to the rapid and uncontrollable creation of convincing false narratives, deepfakes and otherwise misleading synthetic content, making it increasingly difficult for the public to discern fact from falsehood (Norden et al., 2024). Accordingly, the ease with which generative AI can be used to produce and disseminate manipulative content on the internet has amplified the urgency among policymakers, legislators and researchers to devise effective responses. An increasing number of recent initiatives concern developing AI systems that can autonomously detect and counter campaigns of ‘cognitive warfare’ (ELSA Lab Defence, 2022; Van Diggelen et al., 2024; Bateman & Jackson, 2024; Trilateral Research, 2025; Pilati & Venturini, 2025). Solutions range from applications to track and monitor such activities to the active generation of fact-checks or counternarratives or the automatic removal of problematic content from the internet.

This article demonstrates that as a form of epistemic paternalism (Schmechtig, 2025), interventions into the information environment by state authorities have significant implications for citizens’ fundamental rights in liberal-democratic political systems. The use of AI-supported systems to counter instances of ‘cognitive warfare’ likely exacerbates such risks. Further, a review of existing literature in the cognitive and social sciences reveals that research on the impact and harm caused by cognitive warfare campaigns remains inconclusive and contested, as does more recent research on the effects of using generative AI tools for such ends. Therefore, from a legal perspective, any such interventions can only be justified with recourse to the precautionary principle. Given that any measures based on the precautionary principle must be carefully balanced against affected fundamental rights, the article advances the argument that initiatives to use of AI-supported systems against cognitive warfare ought to proceed with utmost caution in light of the characteristics and inherent limitations of such technologies.

Section The concept and realities of ‘Cognitive warfare’ defines ‘cognitive warfare’ and adjacent concepts before assessing the impact AI applications are expected to have on the larger issue of manipulative information online. Section Conceptual clarifications describes solutions that build on machine-learning principles to detect and counter the threat of cognitive warfare. Section ‘Cognitive warfare’ in the age of AI analyses potential conflicts with fundamental rights that such a scenario would entail. Section Utilizing AI to detect and counter ‘Cognitive warfare’ critically assesses the identified issues, based on a review of existing literature on the impact of disinformation and ‘cognitive warfare’, and offers a path to a solution based in law. Section 6 concludes the article.

## The concept and realities of ‘Cognitive warfare’

### Conceptual clarifications

The practice of influencing public opinion within an adversary’s civil society has historical roots as deep as warfare itself. Attempts to shape attitudes and sentiments in foreign populations have been employed to undermine enemies or to convince hesitant allies. With the advent of electronic communication and the rise of dominant social media platforms such as Facebook, TikTok and X, the practice has shifted in important ways. This change coincides with a decline in the relevance of traditional mass media in shaping public opinion in democratic societies (Newman, 2023; Lipka & Shearer, 2023).

The proliferation of false or misleading information now occurs at an unprecedented speed within digitally connected societies. The issue extends beyond electoral manipulation; for instance, during the COVID-19 pandemic, the swift spread of health-related disinformation ostensibly led many individuals to disregard public health guidelines, seek ineffective or harmful treatments, and avoid vaccination efforts (Uscinski & Enders, 2020).

Social media platforms allow for rapid and extensive sharing of content, enabling coordinated influencing efforts to reach vast audiences almost instantaneously. This has facilitated the conduct of information operations, often supported by sophisticated algorithms and data analytics, posing significant challenges to public discourse and democratic processes by hampering citizens’ ability to make informed and facts-based decisions (Bader, 2018). More recent instances, such as the decrease in public support for Ukraine’s defense against Russian aggression, have been linked to orchestrated campaigns directed from Moscow, further highlighting how multi-faceted the problem is (Digital Forensic Research Lab, 2024).

Considering growing geopolitical tensions, the North Atlantic Treaty Organization (NATO) and its Member States have begun using the term ‘cognitive warfare’ to capture the novel dimension of online information as part of a larger, emerging ‘hybrid warfare’ (Bilal, 2021) between democratic, Western societies on one side and an increasing number of authoritarian states on the other. ‘Cognitive warfare’ has been defined in the literature as a non-kinetic form of *warfare* – despite remaining below the threshold of an armed conflict within the meaning of international law – that relies on novel information and communication technologies. Its key feature is the targeting of entire populations with the purpose of changing attitudes and behavior through manipulative communicative techniques (Miller, 2023). The methods employed to generate and disseminate such manipulative content are not mere side effects but essential components of the current, persistent information disorder in Western democratic societies. According to Miller, campaigns of ‘cognitive warfare’ typically attempt to cause two kinds of harm, the first one being psychological (and sometimes physical) harm to individual human beings, the second institutional harm by way of undermining institutional processes and purposes (Miller, 2023, p. 2).

### ‘Cognitive warfare’ in the age of AI

The development of AI technologies, and in particular so-called generative AI, has introduced “an additional layer of complexity” to the threat landscape (Van Diggelen et al., 2024, p. 1). In the wider context of electoral interference, some authors have highlighted the increased threat to democracy posed by the rise of AI-generated synthetic media, such as so-called deepfakes and large language models (LLMs) (Honigberg, 2022; Iyengar, 2024). While some observers argue that concerns are “overblown” (Simon et al., 2023; Simon & Altay, 2025), others suggest that AI-generated content could undermine trust in institutions, manipulate public opinion, and distort political discourse to a degree previously impossible (Barman et al., 2024), through the production of large quantities of content that is both human-like and persuasive (Coombs, 2024).

Growing concerns about deepfake applications, in particular, are based on their ability to portray individuals in misleading ways, thereby manipulating audiences into accepting false narratives and potentially undermining trust. LLMs like ChatGPT can be exploited to create manipulative information and be used for every stage of the disinformation lifecycle (Barman et al., 2024). Other research emphasizes the potential of LLMs to scale and decrease the cost of operations by cutting human labor (Goldstein et al., 2023). Concerns about generative AI are increasingly shared by policymakers (European Commission, 2024).

The literature offers numerous examples of AI-generated content circulating online evidently aimed at the manipulation of political opinion. The development reflects “an ongoing ‘democratization’ of disinformation technologies” (Łabuz & Nehring, 2024, p. 13). One widely cited example concerns the 2023 parliamentary election in Slovakia. Here, a deepfake audio clip seemingly capturing a conversation between pro-EU candidate, Michal Šimečka, and a journalist discussing electoral fraud was circulated just two days before the election, during the country’s media silence period (Meaker, 2023). The case has been interpreted as a paradigmatic example of the vulnerability of democratic processes to generative AI (De Nadal & Jančárik, 2024).

## Utilizing AI to detect and counter ‘Cognitive warfare’

In response to this development, policymakers at the level of the European Union and NATO have begun devising an array of measures to alleviate the peril of ‘cognitive warfare’. Some are legislative. The new EU Digital Services Act (DSA) frames the problem of political disinformation as a “systemic threat” that very large online platforms must address through risk assessment and mitigation measures. The recently issued “Guidelines for Providers of Very Large Online Platforms (VLOPs) and Very Large Online Search Engines (VLOPs)^1^ on the Mitigation of Systemic Risks for Electoral Processes” aim at further strengthening the resilience of political will-formation and democratic decision-making processes within the Union (European Commission, 2024).

Both policymakers and researchers have also begun contemplating AI-supported tools based on the premise that to “achieve the necessary scale and tempo to defend against [AI-generated] threats, utilizing AI as part of the solution seems inevitable” (Van Diggelen et al., 2024, p. 1). To this end, the EU, in particular, has approved public funding for the research and development of a whole range of AI-based tools, frequently through Horizon 2020, the Union’s main research and innovation funding program (Pilati & Venturini, 2025, p. 6). Examples are vera.ai,^2^ ATHENA,^3^ TITAN,^4^ AI4Media,^5^ or AI4Trust.^6^ These and other projects can be divided into two broad categories: (1) algorithms to detect and monitor adversarial campaigns; (2) systems to automatically counter such activities.

Presuming that an effective defense requires awareness of ongoing campaigns and their observation as the basis for decision-making, one concept proposes a “cognitive warfare monitoring and alert system” for NATO agencies (Johns Hopkins University and Imperial College London, 2021). It is intended to utilize machine-learning models capable of detecting adversarial activities through the application of text, image, and video analysis across various online platforms, with the objective of identifying suspicious behavioral patterns. The system could then autonomously track and monitor the progression of these activities and trace their origins (Alizadeh et al., 2020). Continuously produced and updated reports would subsequently inform countermeasures against malicious conduct. Other concepts explore the idea of using machine-learning to identify and analyze problematic pieces of content online more quickly (Bateman & Jackson, 2024, p. 87). Similarly, findings from the ATHENA consortium highlight the utility of AI solutions to detect manipulated or misleading content, to analyze networks of malicious actors, or to classify novel threats (Trilateral Research, 2025). Another of the above-mentioned EU-funded projects, vera.ai, aims at developing verification algorithms to support the work of professional fact-checkers, journalists and media researchers (Ositsyn et al., 2023).

As for the second category, researchers have devised solutions to counter adversarial campaigns with the help of AI-enabled systems. One proposal seeks to utilize AI to de-amplify false and misleading narratives spreading online (Feuerriegel et al., 2023). Another group of researchers has conceptualized a system that can automatically create and distribute factually correct responses to circulating disinformation (He et al., 2023). AI-supported tools could also affix warning labels to potentially algorithmically generated, misleading content or potentially fraudulent digital identities (Goldstein et al., 2023, p. 61). Concerned with user empowerment, one researcher has proposed the design of ‘contextualization engines’ as a possible antidote to misleading information online (Ovadya, 2022). These instruments would work by assisting users in understanding the meaning and significance of content by providing easily accessible context and authoritative information without resorting to censorship. A similar angle is pursued by the EU-funded project TITAN, an AI-supported application that seeks to enable users to acquire critical thinking skills to be able to identify and detect false and misleading content online (TITAN, 2025). As the most invasive algorithmic intervention, van Diggelen et al. have proposed utilizing AI-supported systems to directly respond to ‘cognitive warfare’ activities “by banning or punishing authors or by taking down or labelling messages” as a measure of last resort (Van Diggelen et al., 2024, p. 11).

These ideas are not in themselves new on a conceptual level. Rather, they represent an evolution and adaptation of existing content moderation practices that have been developed and used by major social media platforms for many years. Leading digital platforms have implemented sophisticated machine-learning technologies to detect and neutralize manipulative and misleading activities (Gleicher, 2018). Such automated moderation focuses on filtering out potentially harmful content, with a particular emphasis on disinformation (Gorwa et al., 2020).

Some of the described projects, in contrast, explicitly envision public entities to be in charge of deploying AI-supported systems to detect, monitor, or actively counter campaigns of ‘cognitive warfare’. Examples are the “cognitive warfare monitoring and alert system” to be used by NATO agencies (Johns Hopkins University and Imperial College London, 2021), an “early warning system” against ‘cognitive warfare’ at the disposal of national armed forces (ELSA Lab Defence, 2022), or the national armed forces human-machine team tasked with countering manipulative adversarial conduct in the information space as devised by Van Diggelen et al. (2024). The distinction between such activities carried out by private actors, on the one hand, and by entities with public legal authority, on the other, lies in the potential scope of conduct and the justification for action. Individual digital service companies implement content moderation within their own ecosystems, exercising accountability concerning their specific platforms, often to discharge their legal obligations under applicable frameworks such as the DSA (Husovec, 2024, p. 185).

At least in principle, a state-run application to detect or counter campaigns of ‘cognitive warfare’ would be most effective if it instead monitored online communications across platforms and websites. Although such interventions by both online platforms and state agencies amount to epistemic paternalism – understood as the interference by epistemic authorities “with the activities of other agents without consulting them, in order to make them epistemically better off” (Schmechtig, 2025, p. 1) – in need of justification, private platform governance is based on a contractual relationship between user and company (Klonick, 2017), whereas state intervention by definition is not. For this reason, it not only requires a different legal basis, but also carries different implications from the perspective of users’ fundamental rights (Benedek & Kettemann, 2020). Due to the larger scale and the greater speed with which AI-supported systems can monitor and intervene in online communication, which is indeed advertised as one of the approach’s most distinct advantages (Van Diggelen et al., 2024, p. 1), the implications furthermore differ from state interventions that are entirely human-led. Section 4 addresses these implications.

## Value conflicts

Any intervention by a state against ‘cognitive warfare’ activities can incur considerable costs for fundamental rights. Due to technical limitations in training data and the prevalence of certain biases such as algorithmic, confirmation, or automation biases, risks to fundamental rights may be exacerbated if countermeasures are assisted by AI-supported systems (Shin, 2025; Alon-Barkat & Busuioc, 2023). This section discusses the three rights most immediately affected, which are (1) privacy and data protection; (2) the communication rights of freedom of expression, freedom of information and freedom of the media; (3) and, on a collective level, democratic decision-making and the closely related right to self-determination.

### Privacy and data protection

AI-supported systems are based on data processing. If they are based on the principles of machine learning, they require large amounts of data to learn patterns that, in turn, can be used to identify what they are designed for, such as detecting campaigns of ‘cognitive warfare’. If these systems process personal data, they may interfere with privacy and data protection rights. Typically, false or misleading information online can make use of personal data by discrediting specific people (e.g., X is a foreign agent, Y has committed crimes) or by suggesting that specific people have certain beliefs (e.g., P says it is ok to attack) or act in certain ways (e.g., Q has already attacked). Regardless of such information’s truth value, it can interfere with privacy and data protection rights. If correct, say that Y is indeed a convicted criminal, widely publishing this information may be a (perhaps disproportionate) sanction not imposed by courts. If not correct, it may severely damage his reputation. In both cases, Y may not have control over the information communicated about him.

In the EU, there are two major legal frameworks relevant for privacy and data protection. The fundamental right to privacy can be found in Article 7 of the Charter of Fundamental Rights of the EU (CFEU) and in Article 8 of the European Convention on Human Rights (ECHR). Both rights focus on the protection of private life and family life. The short provisions in this primary legislation are further interpreted in a vast amount of case law. The right to data protection can be found in Article 8 CFEU (Lenaerts, 2012) and is further detailed in secondary legislation in the EU General Data Protection Regulation (GDPR). The GDPR regulates the fair processing of personal data and focuses mainly on data subject rights and data controller obligations. A data subject is the person to whom specific information relates. Data controllers are the entities that have control over the (processing of) personal data. Data subject rights intend to offer data subjects further control over their data and include the right to information (Articles 12–14), the right of access (Article 15), the right to rectification (Article 16), the right to erasure (Article 17), and the right to data portability (Article 18) (Ursic, 2019). Data subjects also have the right not to be subjected to automated decision-making (Articles 21–22 GDPR). Data controller obligations intend to offer further safeguards for data subjects and include the obligation to use data protection by design and by default (Article 25), to keep processing records (Article 30), to cooperate with supervisory authorities (Article 31), to take adequate security measures (Article 32), to notify data breaches (Articles 33–34), to perform impact assessments (Article 35), and to install a data protection officer (Article 37) (Wong, 2021). EU Member States may restrict the application of data subject rights to safeguard defense and national security (Article 23(1)(b) GDPR).

When developing and deploying systems against ‘cognitive warfare’, for the purpose of detecting and monitoring malicious activities in the online information space personal data may be needed on many different types of people, not only adversaries, but also on allies and otherwise uninvolved persons. The reason for this is that the system must be able to make these distinctions, which can only be done by having data on all categories of people and then analyzing what separates these people or groups. Hence, these forms of data processing may interfere not only with privacy and data protection rights of people belonging to an adversary, but also of many others.

Another issue is that any AI system will have error rates, as no system can be a 100% accurate. There may be false positives and false negatives (Custers, 2003). In case of such errors, there can be practical issues (e.g., lack of response or unnecessary response measures), but also justice and fairness issues (e.g., wrong accusations against actors who are not working for an adversary). Such issues may not directly concern privacy or data protection, but they may be caused by harmful or unfair processing of data. Data anonymization and use of distributed systems can in principle reduce these issues (Brunner et al., 2010).

Article 6 of the GDPR requires that there is a legal basis for the processing of personal data. This can be military or public security law containing legal obligations to process the data. However, if the law does not contain such provisions or if private actors are processing personal data, it may be necessary to ask for the consent of data subjects. This can be complicated and undesirable. If people refuse or revoke consent, this may create biased datasets, reducing the accuracy and reliability of early warning systems (Custers et al., 2018).

### Communication rights

Any conceived AI-supported system against campaigns of ‘cognitive warfare’ has significant implications for communication rights. With different degrees of severity, this applies to both those systems that are merely meant to detect and monitor such adversarial behavior and those that automatically counter influence campaigns.

The umbrella concept of communication rights comprises the rights to freedom of expression, freedom of information and freedom of the media. All these rights are protected either explicitly or implicitly by the most important human rights frameworks. On the international level, Article 19 Universal Declaration of Human Rights and Article 19 International Covenant on Civil and Political Rights (ICCPR) protect the right to hold opinions without interference as well as the freedom to seek, receive and impart information and ideas of all kinds. Regionally, Article 10(1) ECHR guarantees the right to freedom of expression, including the freedom to hold opinions and to receive and impart information and ideas without interference by public authority and regardless of frontiers. This right is mirrored in Article 11 CFEU. A rich body of case law and interpretive work by institutions within the United Nations (UN) human rights framework have further specified the contours of the right. It has been emphasized that the scope of freedom of expression covers ideas and information that may “shock, offend or disturb” (European Court of Human Rights, 1976, para. 49) and that it is principally immaterial whether the content of imparted information corresponds with the truth (European Court of Human Rights, 1986, para. 46). As clarified by the UN Human Rights Committee, generally prohibiting expressions of erroneous opinions or incorrect interpretations of past events is not in conformity with the right under the ICCPR (UN Human Rights Committee, 2011, para. 49).

Although closely connected to the right to freedom of expression, the right to freedom of information “is not merely a corollary” but “a right in and of itself” (UN Commission on Human Rights, 2000, para. 42). It is “the touchstone of all the freedoms to which the United Nations is consecrated” (UN General Assembly, 1946). Without it, the entire concept of democratic governance would be inconceivable (UN Commission on Human Rights, 2000, para. 42). Similarly, the media’s “vital role of ‘public watchdog’” (European Court of Human Rights, 1991, para. 59) finds expression in the acknowledgment of freedom of the media as an interrelated yet distinct fundamental right, as clarified by Article 11(2) CFEU.

Communication rights are not without limits. They can be subject to certain restrictions if these meet specific criteria. According to Article 19(3) ICCPR, any restrictions must be provided by law and necessary “[f]or respect of the rights or reputations of others” or “the protection of national security or of public order (*ordre public*), or of public health or morals”. More expansively, Article 10(2) ECHR provides that the communication rights “may be subject to such formalities, conditions, restrictions or penalties as are prescribed by law and are necessary in a democratic society”. However, this is only possible in the interests of national security, territorial integrity or public safety, for the prevention of disorder or crime, for the protection of health or morals, for the protection of the reputation or rights of others, for preventing the disclosure of information received in confidence, or for maintaining the authority and impartiality of the judiciary.

At the same time, given their critical relevance for the functioning of democratic societies, the jurisprudence of the European Court of Human Rights (ECtHR) confirms that the rights themselves must be conceived broadly, while any exceptions to the rights are to be construed narrowly. This is significant in relation to false and misleading information. As a case in point, any laws generally restricting the dissemination of disinformation on the grounds of the information’s untruthfulness *alone* would violate the guarantees of Article 10 ECHR (European Court of Human Rights, 2005, para. 113). Therefore, there must be other factors present to render the restriction of false or misleading information lawful, such as causing damage to another person’s reputation or rights. It is not permitted for a state to restrict disinformation for the purpose of protecting a more indistinct and general concept such as “public harm”, without the information imperiling more tangible values as enumerated in Article 10(2) ECHR (Hoboken, 2019, p. 42).

The guarantees under the fundamental right explicitly apply to information “regardless of frontiers”. While “this does not mean that restrictions may not be imposed on information from abroad, in particular, information that does not make a legitimate contribution to the debate about matters of public concern and is meant to distort the democratic process” (Hoboken, 2019, p. 52), any limitations must be in conformity with the requirements of Article 10(2) ECHR. In the context of ‘cognitive warfare’, several listed purposes present themselves; aside from the safeguarding of ‘national security’ as perhaps the most obvious, state interference with online communication may also aim at preserving public safety or preventing disorder, for example if the adversarial campaign tries to sow societal division through the targeted dissemination of deepfakes depicting racist stereotypes. In a public health emergency such as a global pandemic, the state could invoke the protection of health as a legitimate aim as well. Nevertheless, as a general matter, even if foreign adversaries attempt to influence citizens in Western democracies, any such attempts cannot *wholesale* override the citizens’ own communication rights. It is one of the distinguishing indicators of a liberal democracy that citizens are permitted to choose for themselves what sources of information to consult as the basis for their political will-formation even if those sources distribute false narratives, which is why it is normally not up to democratic governments to paternalistically intervene in the process without invoking clear, demonstrable and unambiguous national security concerns or other legitimate, narrowly circumscribed aims.

The communication rights first and foremost constitute negative freedoms that provide for protection against interference by the state (Bayer et al., 2021, p. 20). An AI-supported system aimed at countering the perceived threat of ‘cognitive warfare’ employed by a state authority would infringe on the rights of freedom of expression and freedom of information because it would essentially enable the state to make distinctions between what content online is ‘good’ and, thus, legitimate, and which is ‘bad’ and could thus be restricted. Especially since the beginning of the COVID pandemic in early 2020, governments in numerous states have started making decisions as to what types of online speech should be considered potentially harmful disinformation and, therefore, be limited (Marecos et al., 2023). Human rights experts have pointed out that this framing has led a number of less democratic and authoritarian governments to exploit the emerging discourse around ‘bad’ information to crack down on communication freedoms more broadly (Kaye, 2020).

Interference with these rights not only occurs once the AI-supported system is tasked with actively countering a campaign of ‘cognitive warfare’, for instance by creating and disseminating counternarratives, blocking accounts or removing content online. Even if it is technically limited to detecting and monitoring an influencing operation, the rights are implicated. Once the algorithmic output predicts online information to form part of such adversarial conduct, human decision-makers will be incentivized to take measures against it. Of course, infringement becomes gradually more severe the more a system proactively manages speech automatically. Depending on the scenario and modes of deployment, on an institutional level such state intervention may infringe on freedom of the media as well, for instance, if algorithmic speech governance is applied to media outlets that are perceived to be part of an ongoing adversarial influence campaign. The fundamental rights implications of such a step would be comparable to those of the ban within the EU of the Russian media organizations RT and Sputnik after the start of Russia’s full-scale invasion of Ukraine in February 2022 (Council of the EU, 2022; Baade, 2022).

### Right to democracy and Self-Determination

Whether the international legal system contains a general ‘right to democracy’ is a contentious issue (Franck, 1992; Klabbers et al., 2021). The assumption of such a legal claim is highly prone to abuse, most importantly in the context of foreign military interventions aiming at regime change (Downes & Monten, 2013). A people’s right to govern themselves is more appropriately captured by the universally recognized principle of self-determination, although its precise outlines remain controversial. The principle is mentioned several times in the Charter of the UN and finds its positive normative expression in Article 1 ICCPR. Aside from this basis in treaty law, it is also recognized as part of customary international law (International Court of Justice, 2024, para. 95).

Its external dimension – both the right to self-governance free from outside interference and the right of a people to form a self-governing entity – is discussed frequently. A coherent and historical interpretation of the principle’s substance recognizes that it further comprises an internal dimension to the effect that any government’s legitimacy is ultimately based on the “consent of the governed” (Lahmann, 2020, p. 203). In that sense, at least where a people have chosen a constitutional arrangement that establishes a form of democratic government, the principle of self-determination protects this choice against both adversarial foreign actors and anti-democratic tendencies of the people’s own government (Fan, 2008). This dimension of self-determination reveals its close relationship to privacy and communication freedoms, which form the *conditio sine qua non* for free public discourse and truly democratic decision-making: first, it is the essence of the right to freedom of expression to bestow citizens with the ability to engage in public discourse to form and shape political opinions for the purpose of participating in the governance of the polity; and second, a lack of privacy will constrain the latitude necessary to exercise expression free from government interference (Bennett & Oduro-Marfo, 2019; Bayer et al., 2021, p. 18). In this context, the principle of self-determination more adequately frames the inherently collective dimension that the individualist fundamental rights can only partly reflect (Lahmann, 2025).

These considerations make clear how the deployment of an AI-based system to either monitor or actively counter a campaign of ‘cognitive warfare’ might infringe on the principle of self-determination as the collective right to democratic decision-making. Despite acknowledging that false and misleading information distorts public political discourse and can amount to a threat to democracy, Lepoutre (2021) convincingly argues that the suppression of such speech might have even more dire systemic consequences because such measures “risk having a severe chilling effect on public discourse” as “people are likely to refrain from making public claims unless they are absolutely sure that these are correct. This, in turn, threatens to stifle many fruitful exchanges – particularly if the give and take of reasons in public discourse is one of our most important tools for *finding out* which views are correct in the first place” (Lepoutre, 2021, p. 112). In other words, even wrong information can contribute to the functioning of a democracy by supporting citizens’ capacity to engage with opinions and values opposed to their own. For this reason, meaningful collective decision-making depends on the freedom to choose the sources of information even if behind those sources, adversarial actors are seeking to influence the recipient on behalf of a foreign power. Liberal democracies require a diverse and heterodox media ecosystem comprising various sources of differing provenance and trustworthiness. Any government intervention with the help of AI to decide what information is acceptable infringes on the process of political will-formation itself and thus implicates the right to self-determination.

## Discussion

As mentioned in Sect. 2.1, campaigns of ‘cognitive warfare’ typically aim at inflicting two kinds of harm, namely psychological (or sometimes physical) damage to individuals and harm to institutions and institutional processes (Miller, 2023, p. 2). It is the expectation of such consequences that justifies state measures amounting to epistemic paternalism because adversaries cannot be allowed to gain “unrestricted access to manipulate the minds of our society using cutting-edge AI technologies” (Van Diggelen et al., 2024, p. 15) and that “[f]ailing to act against adversarial AI-enhanced information warfare poses significant risks” (Coombs, 2024). However, state interventions in the information ecosystems of liberal societies to counter ‘cognitive warfare’ must be carried out without violating citizens’ fundamental rights (Miller, 2023, p. 5). For the interference in these rights to be lawful, it must be *necessary to achieve the legitimate aim* (Gerards, 2013). Therefore, it matters for the lawfulness whether ‘cognitive warfare’ campaigns can in fact cause such harm. Without denying that ‘cognitive warfare’ should be taken seriously as a potential threat, the available research on the subject is reason for caution in this regard. The following section critically assesses the legal and ethical challenges any countermeasures against ‘cognitive warfare’, and especially those utilizing AI-based systems, must account for. The focus is on the connected aspects of causation and attribution.

The scale of the harm caused by campaigns of ‘cognitive warfare’ has remained a fiercely contested question. The main reason for this is the persisting problem to accurately account for the intricate causal mechanics of false and misleading information. The theories that inform the development of countermeasures, either implicitly or explicitly, tend to posit a straightforward causal relationship between the dissemination of misleading information and subsequent harm as the result of altered behavior among the recipients of manipulative messages online. However, the validity of this assumption remains unproven. A majority of experts interviewed as part of the EU-funded ATHENA project conceded that “establishing a direct causal relationship between [cognitive warfare] campaigns and changes in behaviour is one of the most important gaps in the analysis of this phenomenon” (Hytönen et al., 2025, p. 42). Despite the extensive and rapidly expanding body of research exploring the mechanisms behind misleading information and various forms of ‘cognitive warfare’, there is scant empirical evidence to support the hypothesis that such adversarial tactics significantly influence recipients’ behavior (Bateman et al., 2021; Maschmeyer et al., 2025); conversely, some studies suggest the contrary (Mercier & Altay, 2022). One of the issues identified is the lack of consistent longitudinal data to demonstrate, for example, shifts in voting behavior as a consequence of exposure to disinformation campaigns (Hytönen et al., 2025, p. 42).

Recent research has unearthed a large amount of evidence showing that non-democratic adversarial actors have repeatedly endeavored to sway public opinion in Western democratic societies through influence operations. However, it has been pointed out that “evidence of sustained effort is not the same as evidence of impact or prevalence” (Benkler et al., 2018, p. 254). This discrepancy reveals persistent misconceptions regarding the types of information that genuinely affect audience behavior. Rather than being passive recipients of misleading information, susceptible to acting on the false beliefs formed from exposure, individuals actively seek out information that contributes to the systemic coherency of their already established worldview (Arendt, 1958, p. 352). Accordingly, a recipient is likely to be influenced only if a new piece of information – regardless of its veracity – aligns with preexisting convictions within their belief system (Jowett & O’Donnell, 2012, p. 34). This assessment has been demonstrated to generally hold up in the digital information environment: “the causal link between social manipulation and outcomes – beliefs or behavior – is not always straight or linear. A society’s foundation of attitudes, beliefs, and behavior patterns is not subject to easy, direct manipulation. Changing attitudes is hard, and research suggests that the link between attitudes and behavior can be weak” (Mazarr et al., 2019, p. 5).

Similarly, whether the emergence of generative AI is going to increase the effectiveness of ‘cognitive warfare’ campaigns and, thus, exacerbate possible harm remains unconfirmed and has repeatedly been doubted in the literature (Kapoor & Narayanan, 2024). Even accounting for an increasing use of such applications for political ends and the possibility of more sophisticated personalization and micro-targeting (Salvi et al., 2024), first large-scale studies have suggested only modest effects (Simon et al., 2023, p. 3; Simon & Altay, 2025). Such findings align with earlier studies concerning the impact of political influence campaigns on voting behavior, which point to a great number of highly diverse factors contributing to the formation of persistent political beliefs, including, for instance, “familial transmission of political ideology […] rooted in emotional and psychological bonds” that are very resistant to algorithmically generated narratives that contradict them (Elli, 2024). It is such “multiplicity of contextual factors influencing behaviour” that makes it inherently difficult to “isolate the direct behavioural impact” of ‘cognitive warfare’ (Hytönen et al., 2025, p. 42).

The employment of AI-supported technologies does not by itself change the fact that “most exposure [to such content] reinforce[s] existing political beliefs among voters” instead of changing the minds of undecided parts of the electorate (Stockwell, 2024). Only if the latter were achieved would it be justified to conclude that ‘cognitive warfare’ has meaningful effects on electoral outcomes. As Adami contends, “persuasion is difficult, so even high-quality personalised disinformation would likely have a limited impact” (Adami, 2024). The empirical picture to date is inconclusive: One recent large-scale study did provide first evidence that the use of generative AI may indeed amplify the effects of ‘cognitive warfare’ campaigns thanks to expanded possibilities to deploy “personalized persuasion” at scale, i.e., by “matching the content of a persuasive message to the psychological profile of its recipient” (Matz et al., 2024). A different inquiry, however, found that whereas the use of generative AI facilitates the production of larger quantities of misleading content online, AI-generated articles remained on par with human-generated output in terms of persuasiveness (Wack et al., 2025). These results confirm the analysis by Krack et al. (2025) that, in any case, more research is needed to properly evaluate the impact of generative AI on influencing campaigns.

The intricacies of causation can be illustrated by way of two recent cases that have frequently been cited as clear-cut instances of Russian ‘cognitive warfare’ in the guise of electoral interference. The first is the pro-Russian candidate Robert Fico’s victory in the 2023 parliamentary election in Slovakia. His main rival Michal Šimečka’s campaign was thwarted when an audio recording surfaced online that putatively compromised the candidate’s credibility but quickly turned out to have been produced with generative AI. The incident was generally taken in the media as having been a decisive factor for the outcome (Meaker, 2023). However, the picture gets complicated when accounting for additional aspects such as the fact that Russian influence efforts in the country had been established for a long time and that polls carried out ahead of the election revealed that merely 18% of the Slovak electorate expressed trust in their government, components without which the deepfake by itself would probably not have caused any stir in the first place (Nadal & Jančárik, 2024, p. 3). The case suggests that to yield relevant effects at all that might translate into altered voting behavior, campaigns must be sustained and long-term, which in turn makes it inherently difficult to ascribe a particular instance of influencing, like a single deepfake, to a particular outcome.

A closer look at the first round of the 2024 presidential election in Romania similarly calls for caution. While no one could doubt that Russia ran an intensive campaign on TikTok to boost Georgescu’s chances in the run-up to the vote, the larger context reveals that the candidate’s controversial positions could catch on because of “basic socio-economic factors” that had already triggered “massive revulsion against the current establishment and against the main center-left and center-right political parties PSD and the National Liberal Party (PNL) which are perceived as corrupt and self-interested” (Pavel, 2024). Again, drawing a straight causal link between the Russian influence operation and the outcome becomes much less persuasive (Elli, 2024).

The available empirical evidence on the effects of ‘cognitive warfare’, thus, paints a complex picture. Without doubting that various adversarial actors are making persistent and sustained efforts to distort information ecosystems in Western democracies, existing research does not easily support narratives assuming a straightforward causal relationship between such campaigns and undesired outcomes in electoral processes and other contexts (Lahmann, 2022).

A related problem is that of attribution. Several scholars have emphasized that available data shows that many of the most hazardous false political narratives emerge and spread domestically, with adversarial foreign actors merely exploiting and amplifying them (Benkler et al., 2018). In such a case, it can be hard to distinguish between false and misleading information forming part of a campaign of ‘cognitive warfare’ and the quotidian chatter of sensationalist online discourse that often bears hardly any relation to the truth. Even assuming that from a fundamental rights perspective, restrictions may be more easily imposed on information coming from abroad (Hoboken, 2019, p. 52), in many cases disentangling different narratives connected to the same incident, some originating domestically and some abroad, will be impossible. However, the lens of ‘cognitive warfare’ is conducive to such scenario-based solutionism. Often, it will *seem* possible to attribute potentially harmful online speech to a foreign adversarial actor. But even when adversarial conduct can be established, such instances will often be among the clear minority of all the incidents that distort public discourse.

The challenge to empirically establish the harm from ‘cognitive warfare’ due to the problems of causation and attribution has fundamental rights implications. Generally, when a state invokes a legitimate ground for restriction of freedom of expression, “it must establish a direct and immediate connection between the expression and the threat said to exist” (Kaye, 2022, para. 15). When it comes to the idea of countering ‘cognitive warfare’ with the assistance of AI-enabled applications, it is at least doubtful whether a system could be designed that is adequately capable of managing the nuances involved and therefore up for such a complex task. As pointed out in the ethical analysis by some of the approach’s proponents, the risk is high that such a system would flag, and potentially autonomously take down, a large number of false positives, with an additional risk of doing so in a discriminatory manner due to algorithmic biases (Van Diggelen, 2024, p. 13). Even if a system is only used for detection and monitoring, there is a high likelihood that the algorithmic designation of problematic content as forming part of a campaign will activate common human biases such as automation bias or selective adherence (Alon-Barkat & Busuioc, 2023), triggering the resort to aggressive intervention measures that unduly interfere with citizens’ fundamental rights.

It is not argued here that campaigns of ‘cognitive warfare’ do not cause individual or societal harm by affecting recipients’ attitudes and behavior especially as a consequence of long-term exposure but that there remains a lack of demonstrable empirical evidence of causal relationships between campaign and putative effects (Hytönen et al., 2025, p. 42). This complicates the justification of state interventions in online speech, especially with AI-supported systems. However, public law – particularly that of the EU – knows a doctrinal concept that allows for state measures in such situations: the precautionary principle. According to the European Commission, “[r]ecourse to the precautionary principle presupposes that potentially dangerous effects deriving from a phenomenon, product or process have been identified, and that scientific evaluation does not allow the risk to be determined with sufficient certainty” (Commission, 2000, p. 3). Woods and Perrin (2019) have suggested that this fittingly describes the current state of empirical research concerning evidence of harm caused by problematic online speech and thus can serve as an adequate conceptual basis for intervention. Indeed, it has been suggested that the precautionary principle implicitly underlies the risk regime of the EU Digital Services Act which mandates very large online platforms to assess and address possible systemic harm stemming from problematic online speech (Uhlenbusch, 2025; Buchheim, 2022, p. 261) as well as the EU’s approach to AI regulation (European Parliament, 2020, para. 3).

At the same time, Husovec argues against invoking the precautionary principle to address potentially harmful speech online because doing so “can easily slip into giving regulators broad, even if temporary, political powers to regulate speech”, something that in liberal-democratic societies should first and foremost be at the disposal of parliaments but not the executive (Husovec, 2024, p. 280). More generally, Callies has demonstrated that the broad political discretion necessarily attached to assessments and measures based on the precautionary principle inherently carries significant risks to fundamental rights, which is why the measures must have a basis in law and strictly adhere to the proportionality principle (Callies, 2024). As a measure that interferes with fundamental rights can only be proportionate if it is *necessary*, one could envision future legislation that permits *state* intervention in online communication with the assistance of AI-enabled tools to counter a campaign of ‘cognitive warfare’ *if and only if* a competent authority determines the existence of a public crisis in the sense of Article 36 DSA, meaning “extraordinary circumstances [that] lead to a serious threat to public security or public health”. Absent such determination, the online platforms’ own due diligence obligations to manage individual and societal risks stemming from online speech pursuant to the DSA (and similar laws in other countries) should be considered sufficient to fend off any potential threats from adversarial ‘cognitive warfare’ activities, in accordance with the importance of the identified individual rights and values.

## Conclusion

This article has analyzed value conflicts involved in the AI-supported detection and countering of campaigns of ‘cognitive warfare’. While the notion of ‘cognitive warfare’ has long been in use among theorists of military doctrine, with the emergence of digital communications technologies and more recently the development of AI, the threats to open societies from such adversarial practices are perceived to have grown by multiple degrees. The rise of social media has been accompanied by an increasing fragmentation of the information ecosystems in the open societies of Western democracies, a development that adversarial states such as Russia or China have been seeking to exploit for their own geopolitical ends. The approaching ubiquity of LLMs and other forms of AI may further exacerbate the situation.

Against the prevailing narrative, the article has attempted to add a note of caution. The analysis reveals that the problem is less straightforwardly perilous, and more complex and multi-faceted. Any response to ‘cognitive warfare’ inevitably implicates fundamental rights and values such as privacy, communication rights, and self-determination. Therefore, policymakers are advised to focus on measures that preserve these rights, rather than resorting to seemingly quick technological fixes (Hoboken, 2019, p. 42). Given the lack of firm empirical evidence clearly demonstrating the causal mechanics of ‘cognitive warfare’, considerations based on the precautionary principle might point to a way forward if grounded in the rule of law, including strict application of proportionality. Such an approach would maintain the balance necessary to foster the rights and values that are essential for sustaining open and democratic societies in the age of information disorder.

## Data Availability

No datasets were generated or analysed during the current study.

## Footnotes

1 According to Article 33(1) DSA, VLOPs and VLOSEs are online platforms and search engines with more than 45 million active monthly users within the EU.

2
https://www.veraai.eu/home.

3
https://project-athena.eu/.

4
https://www.titanthinking.eu/objectives.

5
https://www.ai4media.eu/project-overview/.

6
https://cordis.europa.eu/project/id/101070190.
